# First-in-human evaluation of [^18^F]-AlF-NOTA-neurotensin for NTSR1-targeted imaging of prostate cancer: a head-to-head comparison with [^68^Ga]Ga-PSMA-617

**DOI:** 10.1080/07853890.2026.2678019

**Published:** 2026-06-17

**Authors:** Jiawei He, Yinzhao Wang, Wei Tang, Xiaomei Gao, Yi Cai, Yongxiang Tang, Shuo Hu

**Affiliations:** ^a^ Department of Urology, Disorders of Prostate Cancer Multidisciplinary Team, National Clinical Research Center for Geriatric Disorders, Xiangya Hospital Central South University, Changsha City, Hunan Province, P.R. China; ^b^ Department of Pathology, Disorders of Prostate Cancer Multidisciplinary Team, National Clinical Research Center for Geriatric Disorders, Xiangya Hospital Central South University, Changsha City, Hunan Province, P.R. China; ^c^ Department of Nuclear Medicine, National Clinical Research Center for Geriatric Disorders, Key Laboratory of Biological Nanotechnology of National Health Commission, Xiangya Hospital Central South University, Changsha City, Hunan Province, P.R. China

**Keywords:** Prostate cancer, PSMA, NTSR1, PET/CT

## Abstract

**Background:**

Neurotensin receptor-1 (NTSR1) is a promising target for prostate cancer imaging. This study evaluates the first-in-human NTSR1-directed radiotracer [^18^F]-AlF-NOTA-neurotensin and compares it with [^68^Ga]Ga-PSMA-617.

**Methods:**

In a single-centre, prospective study, 23 men with biopsy-verified prostate cancer were enrolled at Xiangya Hospital (June 2020–June 2024). Within 7 days before radical prostatectomy, whole-body PET/CT was performed after sequential administration of [^68^Ga]Ga-PSMA-617 and [^18^F]-AlF-NOTA-neurotensin. Imaging metrics were assessed against histopathology. Immunohistochemistry (IHC) for PSMA and NTSR1 was conducted to correlate tracer uptake with receptor expression.

**Results:**

The median age was 71 years (IQR 66–75); 14 were treatment-naïve and 9 had prior androgen-deprivation therapy (ADT). In untreated patients, [^68^Ga]Ga-PSMA-617 showed superior sensitivity (92.9%), specificity (100%), PPV (100%), and NPV (93.3%) compared to [^18^F]-AlF-NOTA-neurotensin . At the lesion level, [^68^Ga]Ga-PSMA-617 localized 36 of 38 foci (94.7%), while [^18^F]-AlF-NOTA-neurotensin identified none (0%; *p* < 0.05). After short-term ADT, [^68^Ga]Ga-PSMA-617 sensitivity dropped to 33.3%, while [^18^F]-AlF-NOTA-neurotensin sensitivity rose to 77.8% (*p* < 0.05). Lesion-based detection rates were 36% and 64%, respectively, and there were no significant differences in specificity, PPV, or NPV. IHC revealed a significant decrease in PSMA H-score from 9 (IQR 6.5–12) to 7 (IQR 1.3–8.8; *p* = 0.034) and an increase in NTSR1 H-score from 1 (IQR 0.25–2) to 6 (IQR 3–8; *p* = 0.003).

**Conclusion:**

[^68^Ga]Ga-PSMA-617 remains the gold standard for treatment-naïve prostate cancer, whereas [^18^F]-AlF-NOTA-neurotensin provides complementary-diagnostic value by enhancing lesion detection after ADT. Our findings support a stage-adapted, receptor-driven imaging strategy that addresses tumor phenotypic heterogeneity, enabling more precise management throughout the disease course.

## KEY MESSAGES

- First-in-human evaluation of NTSR1-targeted PET tracer [^18^F]-AlF-NOTA-neurotensin for prostate cancer.
- [^68^Ga]Ga-PSMA-617 demonstrates 92.9% sensitivity pre-ADT and outperforms [^18^F]-AlF-NOTA-neurotensin.
- After short-term ADT, [^18^F]-AlF-NOTA-neurotensin sensitivity increases to 77.8%, surpassing [^68^Ga]Ga-PSMA-617.
- IHC confirms PSMA downregulation and NTSR1 upregulation following short-term androgen deprivation.
- A receptor-expression-guided, stage-adapted PET imaging strategy proposed for precision management of prostate cancer.

## Introduction

Prostate cancer (PC) is the second most common malignancy in males and the fifth leading cause of cancer-related death among males [1]. Androgen-deprivation therapy (ADT) combined with androgen receptor signaling inhibitors (ARSI) provides benefits for metastatic disease or for down-staging prior to radical surgery [2]. After 14–20 months of endocrine treatment, however, a subset of patients develop therapeutic resistance, tumour recurrence and metastasis, as their disease progresses from castration-sensitive prostate cancer (CSPC) to castration-resistant prostate cancer (CRPC) [3]; at this stage, tumours no longer respond to hormonal regimens, and the 5-year survival rate is in the range of 15–36% [4,5].

Prostate-specific membrane antigen (PSMA) is highly expressed in most prostate cancer cells and correlates with higher prostate-specific antigen (PSA) values and higher International Society of Urological Pathology (ISUP) grade at diagnosis [6,7]; consequently, several PSMA-based radiotracers are now used routinely in the clinic. [^68^Ga]Ga-PSMA-617, a novel PSMA-targeted tracer, is valuable for primary tumour detection and initial staging [8]. In advanced disease or CRPC, however, PSMA expression may be lost [9], rendering PSMA PET unreliable for the management of PSMA-negative prostate cancer; tumours may progress while being undetected. This necessitates novel targets to complement treatment-response monitoring and enable comprehensive lesion detection.

Neurotensin receptor 1 (NTSR1) is the high-affinity receptor for neurotensin (NTS) [10] and is overexpressed in breast [11], colorectal [12] and pancreatic cancers [13,14]. Our previous tissue-based study showed that >90% of prostate cancer specimens displayed high or moderate NTSR1 expression—higher than the 86.7% PSMA positivity rate—and that all PSMA-negative tissues were NTSR1-positive, suggesting that NTSR1-targeted imaging or therapy could complement PSMA-targeted approaches [15]. The potential of NTSR1 as a theranostic target has prompted the development of several promising radioligands, which have been rigorously evaluated in preclinical settings [16,17]. While these probes have demonstrated high binding affinity and specificity in laboratory and animal models [18], their clinical utility and diagnostic efficacy in human subjects have yet to be established. Building upon our previous histopathological findings, we hypothesized that NTSR1-targeted PET imaging could offer a significant strategic advantage, particularly in detecting PSMA-negative lesions. Consequently, we developed [^18^F]-AlF-NOTA-neurotensin, a tracer engineered for high-affinity uptake in NTSR1-positive tumors. In this study, we conducted a first-in-human clinical evaluation of [^18^F]-AlF-NOTA-neurotensin in patients with prostate cancer to determine its potential as a complementary modality to PSMA PET, aiming to achieve comprehensive lesion detection and improved treatment monitoring in the context of PSMA-negative disease or CRPC.

## Materials and methods

### Patient selection

This single-centre, prospective, head-to-head cohort study was approved by the Ethics Committee of Xiangya Hospital (Approval Number: 201801002) and registered at ClinicalTrials.gov (NCT03516045). Patients with histopathologically confirmed prostate cancer were enrolled between June 2020 and June 2024 after being diagnosed by needle biopsy. Individuals who could not undergo PET imaging, those for whom the interval between the two tracer imaging sessions exceeded 15 days, and patients for whom prostate cancer had been diagnosed and treated prior to enrollment were excluded. All enrolled patients received both [^68^Ga]Ga-PSMA-617 and [^18^F]-AlF-NOTA-neurotensin before radical prostatectomy (RP). Clinical data—including age, PSA level, PET/CT findings, clinical T stage (cT), and pathological results (Gleason score and ISUP grade)—were collected for every participant.

### Radiochemistry

[^68^Ga]Ga-PSMA-617 was prepared as described previously [19]. For the neurotensin tracer, the radiolabeling procedure was based on an optimized [^18^F]AlF-complexation method [20]. Briefly, AlCl_3_ (2 mM, 3 μL) was pre-incubated with the NOTA-neurotensin precursor (2 mM, 6 μL) in a sodium acetate buffer (1.0 M, pH 4.0–4.5). Subsequently, 20 μL of aqueous [^18^F]fluoride (0.25–0.40 GBq) and 100 μL of acetonitrile were introduced into the mixture. The reaction was conducted in a closed system at 100 °C for 10 min. Upon completion, the solution was cooled to ambient temperature and quenched with 5% acetic acid. To ensure high radiochemical purity, the crude mixture was passed through an alumina cartridge to remove unreacted [^18^F]fluoride, followed by purification *via* semi-preparative HPLC. The fraction corresponding to the product (retention time: ∼8.1 min) was collected, trapped on a C18 cartridge to remove organic solvents and salts, eluted with ethanol, and finally formulated in sterile 0.9% NaCl solution for clinical administration.

Preparation and quality control were performed at the PET Centre of Xiangya Hospital; radiochemical purity was >95% for both tracers. PET/CT imaging was carried out on a Discovery PET/CT scanner (GE Healthcare, USA). After obtaining written informed consent, patients underwent whole-body PET/CT imaging targeting NTSR1 and PSMA; The two examinations were completed within 15 days. Each tracer was injected intravenously at 0.1 mCi kg^−1^. For [^18^F]-AlF-NOTA-neurotensin, the total mass of the peptide injected per patient was strictly maintained between 1.8 μg and 4.2 μg (mean 3.1 ± 0.9 μg). By maintaining a high specific activity, it was ensured that the tracer administration was well below the saturation threshold for NTSR1 receptors, thereby avoiding any potential pharmacological side effects or competitive receptor inhibition. This was followed by a 60-min resting period before acquisition from skull base to mid-thigh and of a separate brain scan. CT was performed first (120 kV, 200 mA, 3 mm slice thickness), followed by PET (2 min per bed position for torso, 4 min for brain, 3 mm slice thickness). Transaxial, coronal and sagittal PET, CT and fused PET/CT images were generated by iterative reconstruction and analysed by two nuclear medicine physicians with PET/CT experience. Diagnoses were based on visual assessment and quantitative parameters such as maximum standardized uptake value (SUVmax). Each scan was interpreted independently; discrepancies were resolved by consensus after discussion.

To ensure accurate spatial co-registration between PET ‘hot spots’ and histopathological nodules, a standardized alignment protocol was implemented [21]. Following radical prostatectomy, specimens were fixed in 10% buffered formalin and step-sectioned at 3 mm intervals from the apex to the base, in a plane perpendicular to the prostatic urethra. This orientation was maintained to align with the axial plane of the PET/CT scans. Anatomical markers, including the urethra, prostatic capsule, and seminal vesicles, served as reference points for alignment. A nuclear medicine physician and a urologic pathologist then performed a consensus-based review, mapping PET-positive lesions to specific ISUP-graded nodules by correlating, between the imaging slices and the pathological slides, their relative anatomical quadrants and distances from the apex or base.

### Immunohistochemical (IHC) staining and analysis

The IHC staining of the tissues was performed and analyzed by two experienced urologic pathologists. Briefly, the mouse anti-human PSMA (1:100) and rabbit anti-human NTSR1 (1:100) antibodies were used as the primary antibodies for the IHC staining of PSMA and NTSR1, respectively. The images of three representative fields were captured using Leica Qwin Plus v3 software at 200× magnification. Three fields within the tumor were randomly selected, and staining intensity was scored. Tissues with staining intensity equivalent to background; pale-yellow, yellow, and brown staining received 1, 2, and 3 points, respectively. The number of positive cells in each field was counted and scored according to the percentage of positivity: ≤5% = 0; 6–25% = 1; 26–50% = 2; 51–75% = 3; >75% = 4. The intensity score and the percentage score were multiplied; the final value was graded as negative (–) at 0, weak positive (+) for 1–4, moderate positive (++) for 5–8, and strong positive (+++) for ≥9.

### Statistical analysis

All analyses were performed with IBM SPSS 25.0.0 (IBM Corp., Armonk, NY, USA). Continuous variables are presented as median(IQR) and compared by Mann–Whitney U or t-test; categorical variables are expressed as number (percentage) and compared by χ^2^ test. Owing to the small number of discordant pairs, conventional exact McNemar tests were underpowered; therefore, paired bootstrap resampling was used to estimate confidence intervals for differences in sensitivity, specificity, positive predictive value and negative predictive value between the two probes. The 2.5th and 97.5th percentiles of the bootstrap distribution were taken as the 95% CI; if the interval did not contain 0, the difference was considered statistically significant. *p* < 0.05 was regarded as significant.

## Results

### Overall clinicopathological characteristics of the enrolled patients

A total of 23 prostate cancer patients were enrolled, all of whom ultimately underwent radical prostatectomy. Fourteen patients proceeded directly to surgery after pathological diagnosis, yielding 38 lesions; the remaining 9 received a median of 4 months (range, 3–6 months; Supplementary Table 1) of ADT due to clinical requirements before undergoing radical prostatectomy, yielding 25 lesions. Detailed clinical data, tumour stage, ISUP grade and pathological information for both groups are summarized in Table 1.

**Table 1.** Clinical and pathological characteristics of prostate cancer in treatment-naïve patients and in those after androgen-deprivation therapy (ADT).

Variable	Treatment-naïve	After ADT
**Age (years)**	68(64-74), *n* = 14	64(64-73), *n* = 9
**BMI(kg/m^2^)**	22(21-24), *n* = 11	22(20-23), *n* = 6
**DRE (cases)**		
Positive	4(33.3%)	3(50%)
Negative	8(66.7%)	3(50%)
**PSA level**		
TPSA (ng/mL)	15(7.4-36), *n* = 14	38(8-87), *n* = 9
FPSA (ng/mL)	0.96(0.47-4.3), *n* = 11	5.5(2.9-8.4), *n* = 4
F/TPSA	0.09(0.064-0.14), *n* = 11	0.10(0.067-0.13), *n* = 4
**mpMRI (cases)**		
Positive	8(66.7%)	3(75%)
Negative	4(33.3%)	1(25%)
**[^68^Ga]Ga-PSMA-617**		
Positive (cases)	13(92.9%)	3(33.3%)
Negative (cases)	1(7.1%)	6(66.7%)
Maximum SUV of suspicious (main) lesions	19(12-27), *n* = 13	8.8(5.8-10), *n* = 3
**[^18^F]-AlF-NOTA-neurotensin**		
Positive (cases)	0(0%)	6(66.7%)
Negative (cases)	14(100%)	3(33.3%)
Maximum SUV of suspicious (main) lesions	–	17(16-17), *n* = 6
**Pathological lesions**	38	25
**Pathological examination results (cases)**		
**Tumor staging**		
pT2	8(66.7%)	1(12.5%)
pT3	3(25%)	3(37.5%)
pT4	1(8.3%)	4(50%)
**ISUP grade group**		
1	1(8.3%)	–
2	2(16.7%)	–
3	2(16.7%)	–
4	3(25%)	–
5	4(33.3%)	–
**Invasion of capsule**	6(42.9%)	8(88.9%)
**Invasion of seminal vesicles**	1(7.1%)	5(55.6%)
**Regional lymph node metastasis**		
pN0	13(92.9%)	7(77.8%)
pN1	1(7.1%)	2(22.2%)

BMI = Body Mass Index; DRE = Digital rectal examination; TPSA = Total PSA; FPSA = Free PSA; F/TPSA = Free PSA/Total PSA.

### Diagnostic performance of [^68^Ga]Ga-PSMA-617 and [^18^F]-AlF-NOTA-neurotensin in treatment-naïve prostate cancer

Detailed patient-level and lesion-level results are presented in Table 2. Among the 14 patients who had not received ADT, [^68^Ga]Ga-PSMA-617 showed a per-patient sensitivity of 92.9% (95% CI 64.2%–99.6%), significantly higher than the sensitivity of 0% (95% CI 0%–26.8%) obtained with [^18^F]-AlF-NOTA-neurotensin (*p* < 0.05).

**Table 2.** Comparative evaluation of the diagnostic efficacy of two molecular imaging probes in treatment-naïve patients.

Type of PET/CT	Diagnostic efficacy of treatment-naïve prostate cancer			
	Sensitivity	Specificity	PPV	NPV
[^68^Ga]Ga-PSMA-617	92.9%(95%CI: 64.2%-99.6%)	100%(95%CI: 73.2%-100%)	100%(95%CI: 71.7%-100%)	93.3%(95%CI: 66%-99.7%)
[^18^F]-AlF-NOTA-neurotensin	0%(95%CI: 0%-26.8%)	92.9%(95%CI: 64.2%-99.6%)	0%(95%CI: 0%-94.5%)	48.1%(95%CI: 29.2%-67.6%)

PPV = Positive Predictive Value; NPV = negative predictive value.

Differences in specificity (100% vs 92.9%, *p* = 0.80) and negative predictive value (93.3% vs 48.1%, *p* = 0.32) were not statistically significant. At the lesion level, [^68^Ga]Ga-PSMA-617 correctly identified 36 of 38 malignant foci (detection rate: 94.7%), with only two lesions missed; [^18^F]-AlF-NOTA-neurotensin detected none (0/38, detection rate 0%). The difference in detection rate was significant (*p* < 0.05), corresponding to an relative reduction in missed lesions of 94.7%.

**Figure 1.**
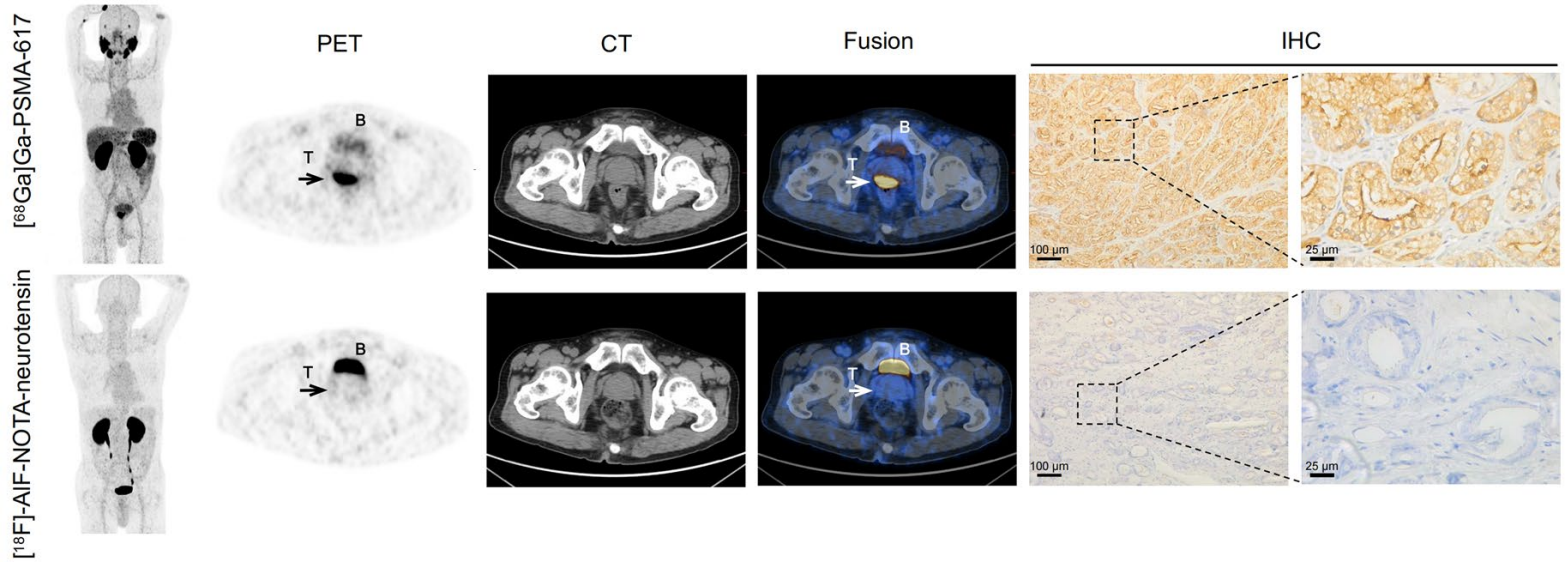
Representative [^68^Ga]Ga-PSMA-617 PET/CT and [^18^F]-AlF-NOTA-neurotensin PET/CT images of a lesion in a treatment-naïve prostate-cancer patient, together with corresponding immunohistochemical staining. The lesion was clearly positive on [^68^Ga]Ga-PSMA-617 PET/CT and showed concordant PSMA positivity on immunohistochemistry; it was negative on [^18^F]-AlF-NOTA-neurotensin PET/CT, with correspondingly negative NTSR1 staining. T: tumor; B: bladder. Scale bars are shown.

Figure 1 illustrates representative images from one treatment-naïve patient: the lesion was clearly positive on [^68^Ga]Ga-PSMA-617 PET and showed concordant PSMA expression on immunostaining, whereas it was negative on [^18^F]-AlF-NOTA-neurotensin PET with correspondingly negative NTSR1 staining. Thus, for treatment-naïve prostate cancer, [^18^F]-AlF-NOTA-neurotensin offers no diagnostic value, while [^68^Ga]Ga-PSMA-617 maintains excellent sensitivity.This obviates the need for additional NTSR1-targeted imaging.

### Diagnostic performance of [^68^Ga]Ga-PSMA-617 and [^18^F]-AlF-NOTA-neurotensin in prostate cancer after ADT

Detailed results are presented in Table 3. After ADT (*n* = 9), the sensitivity of [^18^F]-AlF-NOTA-neurotensin was significantly higher than that of [^68^Ga]Ga-PSMA-617 (77.8% vs. 33.3%, *p* < 0.05). Differences in specificity (66.7% vs. 77.8%), positive predictive value (70% vs. 60%), and negative predictive value (75% vs. 53.8%) did not reach statistical significance (all *p* > 0.05). At the lesion level (*n* = 25 foci), [^18^F]-AlF-NOTA-neurotensin demonstrated a significantly higher detection rate compared to [^68^Ga]Ga-PSMA-617 (64% [16/25] vs. 36% [9/25], *p* = 0.012). Notably, [^18^F]-AlF-NOTA-neurotensin successfully identified 11 additional lesions that were missed by PSMA PET, reducing the number of PSMA-missed lesions that remained undetected from 16 to 5 (a 68.8% reduction in PSMA-related missed foci, or a 44% reduction in overall miss rate relative to the total 25 lesions).

**Table 3.** Comparative evaluation of the diagnostic efficacy of two molecular imaging probes in patients after androgen-deprivation therapy.

Type of PET/CT	Diagnostic efficacy of after androgen-deprivation therapy prostate cancer			
	Sensitivity	Specificity	PPV	NPV
[^68^Ga]Ga-PSMA-617	33.3%(95%CI: 9%-69.1%)	77.8%(95%CI: 40.2%-96%)	60%(95%CI: 17%-92.7%)	53.8%(95%CI: 26.1%-79.6%)
[^18^F]-AlF-NOTA-neurotensin	77.8%(95%CI: 40.2%-96.1%)	66.7%(95%CI: 30.9%-90.9%)	70%(95%CI: 35.3%-91.9%)	75.0%(95%CI: 35.6%-95.5%)

PPV = Positive Predictive Value; NPV = negative predictive value.

**Figure 2.**
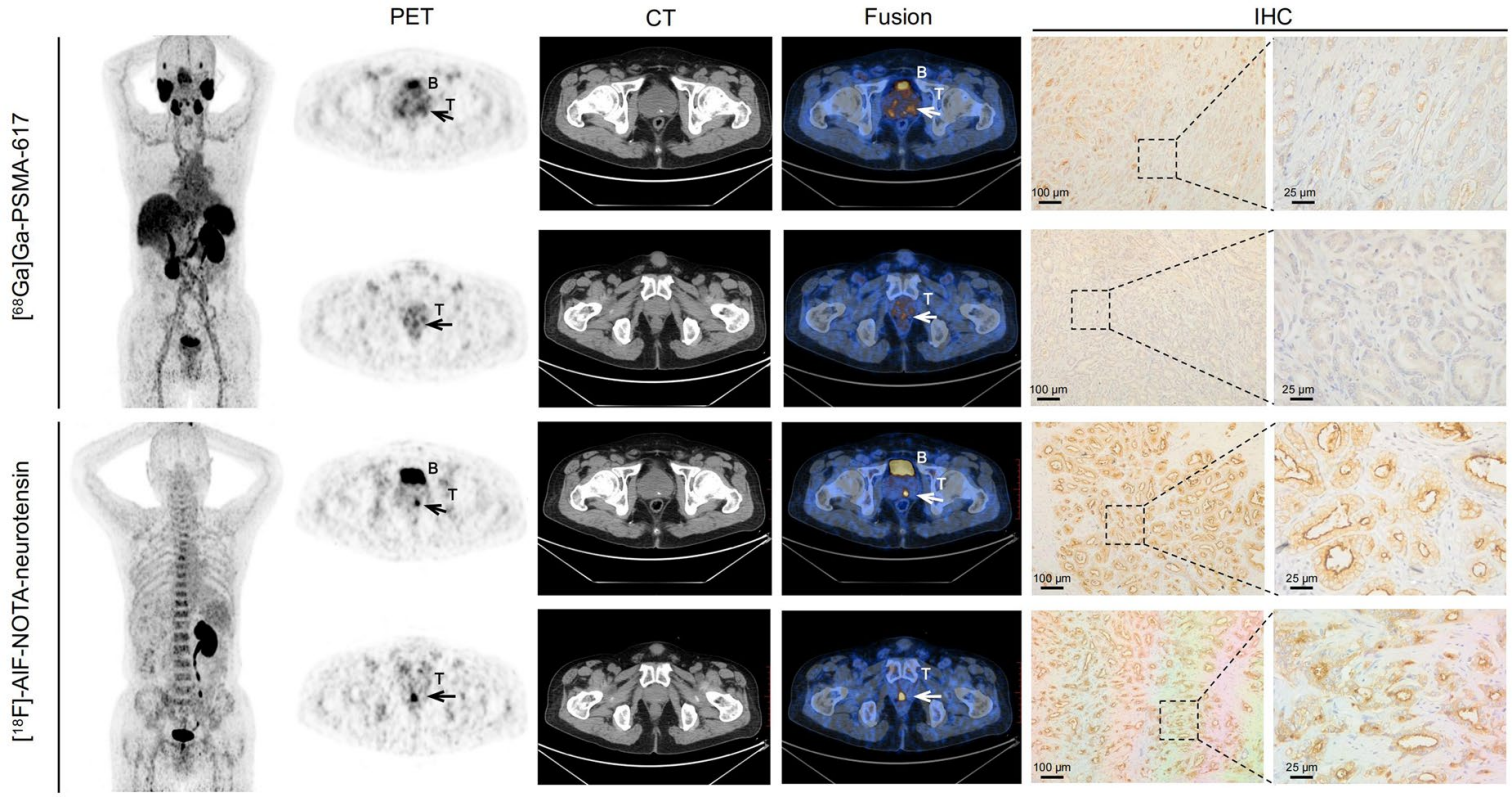
Representative [^68^Ga]Ga-PSMA-617 PET/CT and [^18^F]-AlF-NOTA-neurotensin PET/CT images of two lesions in a patient after 4 months of ADT, together with corresponding immunohistochemical staining. One lesion was located posterior to the prostatic apex and demonstrated focal uptake of the neurotensin tracer (SUVmax 16.4); the other was in the left posterior central gland (SUVmax 10.6). Both lesions were negative on [^68^Ga]Ga-PSMA-617 PET/CT. Immunohistochemistry revealed negative or weakly positive PSMA staining,with positive NTSR1 staining. T: tumor; B: bladder. Scale bars are shown.

A representative patient received 4 months of ADT; two lesions were identified by [^18^F]-AlF-NOTA-neurotensin, one located posterior to the prostatic apex (SUVmax16.4) and the other located in the left posterior central gland (SUVmax10.6). Notably, these lesions were negative on [^68^Ga]Ga-PSMA-617 PET. Immunohistochemistry of the two lesions revealed negative or weakly positive PSMA staining and positive NTSR1 staining (Figure 2).

Thus, in the setting of biochemical recurrence after ADT, [^18^F]-AlF-NOTA-neurotensin can serve as an effective complementary probe for PSMA-negative lesions, offering higher patient-level sensitivity and a higher lesion-level detection rate without compromising specificity.

### Changes in PSMA and NTSR1 expression levels before and after ADT

**Figure 3.**
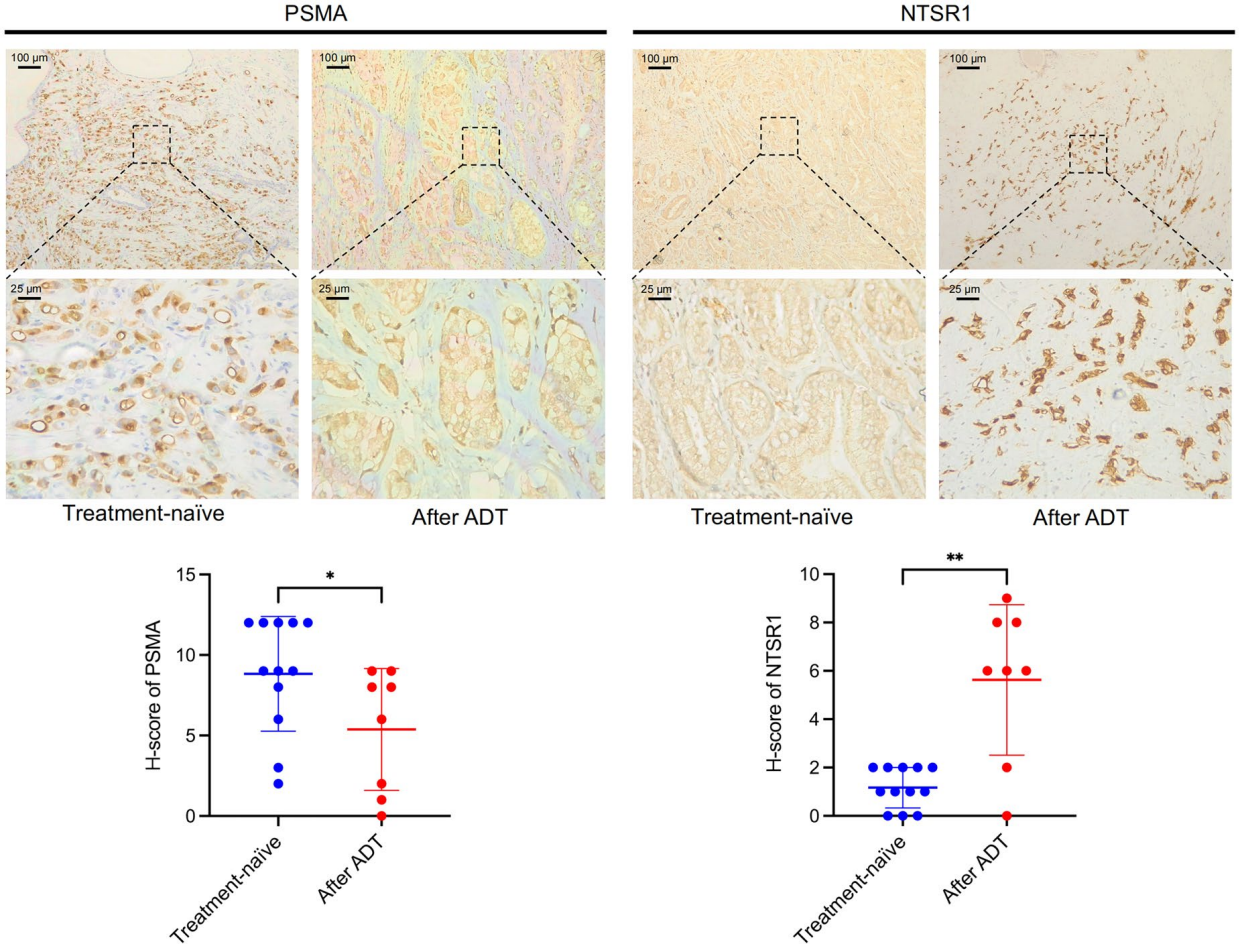
Representative immunohistochemical images and statistical analysis of PSMA and NTSR1 expression in primary lesions from 12 treatment-naïve prostate-cancer patients and 8 after ADT patients. Scale bars are shown.

To verify whether changes in PSMA and NTSR1 expression before and after ADT correlate with the uptake trends of the two tracers, immunohistochemical staining was performed on the primary lesions of 12 prostate cancer patients who had not received ADT and 8 who had. The results showed that the median IHC score for PSMA in treatment-naïve prostate cancer was 9 (IQR 6.5–12), whereas it significantly decreased to 7 (IQR 1.3–8.8) after ADT (*p* = 0.034). The median IHC score for NTSR1 in treatment-naïve prostate cancer was 1 (IQR 0.25–2), whereas it significantly increased to 6 (IQR 3–8) after ADT (*p* = 0.003) (Figure 3).

## Discussion

Monitoring therapeutic response in prostate cancer remains challenging because the timing of resistance and metastasis is unpredictable [22]. The high specificity and sensitivity of prostate-specific membrane antigen (PSMA) in prostate cancer have made it an effective imaging and therapeutic target; PSMA PET is recommended for primary staging and follow-up during the hormone-sensitive phase [23], and [^177^Lu]Lu-PSMA-617 has recently been approved for metastatic castration-resistant prostate cancer (CRPC) [24]. However, as tumours advance to late or resistant stages, PSMA expression becomes heterogeneous and variable, and is lost in 15–20% of CRPC cases, magnifying the limitations of PSMA PET in PSMA-negative patients [25].

Our previous study showed that all PSMA-negative prostate cancer tissues expressed NTSR1, with 80% showing high expression; in relatively early disease (patients without evidence of recurrence or metastasis after ADT), NTSR1 expression was evenly distributed among strong, moderate, weak and negative categories, whereas in advanced and metastatic disease NTSR1 expression was uniformly strong [15]. This suggests a complementary relationship between NTSR1 and PSMA.

In this head-to-head human study, [^68^Ga]Ga-PSMA-617 achieved a patient-level sensitivity of 92.9% (95% CI 64.2%–99.6%) and specificity of 100% (95% CI 73.2%–100%) in treatment-naïve patients, with a lesion detection rate of 94.7% (36/38). These results are consistent with previous reports and confirm PSMA PET as the preferred modality for initial diagnosis and staging [26]. In contrast, [^18^F]-AlF-NOTA-neurotensin showed a sensitivity of 0% (95% CI 0%–26.8%) and specificity of 92.9% (95% CI 64.2%–99.6%), missing all 38 lesions. We propose that this result reflects a specific biological state rather than a deficiency in tracer performance. Considering that [^18^F]F-AlF-NOTA-neurotensin has demonstrated high binding affinity for NTSR1 in preclinical models [18], our IHC data—showing minimal NTSR1 expression in treatment-naïve prostate cancer (median H-score = 1)—suggest that while the receptors are present, their density remains below the intrinsic detection threshold of PET imaging. We interpret this as evidence of a reciprocal expression pattern between the two markers, where NTSR1 predominantly surfaces in PSMA-negative tumors [27]; this biological silence provides a distinct baseline for untreated prostate cancer, which further underscores the clinical significance of the dramatic surge in NTSR1-PET sensitivity observed following ADT.

The biological mechanisms underlying the differential expression of NTSR1 can be attributed to the transition of oncogenic drivers and lineage plasticity under therapeutic pressure [28]. In the treatment-naïve stage, the AR signaling pathway serves as the primary driver of tumor growth; while AR signaling positively regulates PSMA expression, it typically exerts a suppressive effect on alternative pro-survival markers, including NTSR1. However, under sustained selective pressure from ADT, tumor cells may undergo a phenotypic switch to evade apoptosis and bypass AR blockade, transitioning from a luminal adenocarcinoma phenotype toward AR-independent lineages.

This lineage reprogramming enables tumor cells to maintain sustained proliferation *via* non-AR pathways, a process often accompanied by a profound downregulation or complete loss of PSMA expression. Our cohort data support this biological transition: even after short-term intervention, the diagnostic efficacy of [^68^Ga]Ga-PSMA-617 declined significantly to a sensitivity of 33.3%, paralleled by a significant decrease in PSMA IHC scores (median score from 9 to 7, *p* = 0.034). Conversely, [^18^F]-AlF-NOTA-neurotensin sensitivity rose dramatically to 77.8%, with IHC confirming a concurrent and significant increase in NTSR1 expression.

Upon activation, the binding of neurotensin to NTSR1 triggers a cascade of downstream pro-survival signaling pathways, including the activation of the mitogen-activated protein kinase (MAPK) and Phosphatidylinositol-3-kinase (PI3K) pathways, as well as the phosphorylation of epidermal growth factor receptor (EGFR), Src, and STAT5 [29–31]. These molecular events collectively enhance tumor DNA synthesis and bolster cell survival in an androgen-depleted environment.

The identification of several lesions by [^18^F]-AlF-NOTA-neurotensin that were entirely missed by [^68^Ga]Ga-PSMA-617 further underscores the dynamic and heterogeneous nature of PSMA expression under ADT and during tumor evolution. Previous studies have consistently highlighted the complexity of PSMA fluctuations following ADT [21]. For instance, Afshar-Oromieh et al. [32] observed that over a median of 230 days of ADT, 71% of patients showed a decrease in mean tracer uptake, while 12.9% of lesions exhibited an increase. Similarly, Emmett et al. [33] demonstrated that although LHRH agonists combined with bicalutamide rapidly reduced median PSMA uptake in CSPC, significant inter-patient heterogeneity persisted, likely reflecting individual variations in therapeutic response. Furthermore, Ettala et al. [34] noted a transient increase in PSMA uptake after short-term (3–4 weeks) ADT, followed by a gradual decline as treatment progressed. Our results expand upon these observations by demonstrating that during the specific biological window in which PSMA signals are attenuated or rendered heterogeneous, NTSR1 emerges as a robust compensatory biomarker, ensuring the reliable detection of aggressive or treatment-resistant tumor clones.

Consequently, the synergistic use of [^68^Ga]Ga-PSMA-617 and [^18^F]-AlF-NOTA-neurotensin may provide more comprehensive diagnostic and follow-up utility by capturing the diverse molecular phenotypes present across the evolutionary spectrum of prostate cancer.

Beyond its diagnostic utility, the robust expression of NTSR1 in post-ADT tumors opens new avenues for targeted therapeutic interventions. NTSR1-targeted radioligand Therapy (RLT), utilizing isotopes such as ^177^Lu or ^225^Ac, represents a promising strategy for patients who develop resistance to conventional AR-targeted therapies or those with PSMA-negative disease. Furthermore, a combined or sequential targeting strategy (integrating both PSMA and NTSR1) could address the inherent spatial and temporal heterogeneity of prostate cancer, minimizing the risk of ‘tumor escape’ that occurs with single-antigen-targeted treatments. In the post-ADT landscape, where PSMA expression may be heterogeneously suppressed, NTSR1 PET provides a ‘molecular backup’, ensuring that aggressive, treatment-resistant clones are not overlooked. This allows for more refined patient stratification, guiding clinicians to switch from AR-directed therapies to NTSR1-targeted or other systemic treatments at the earliest sign of phenotypic transformation.

Limitations include the single-centre design and limited sample size. Specific ADT agents were not distinguished, and their differential effects on PSMA and NTSR1 expression remain unclear. Due to the categorical nature of the data and the small number of positive cases, this study focused on group-level shifts rather than a lesion-to-lesion correlation analysis. Finally, the study was limited to hormone-sensitive disease. Future studies will focus on CRPC and rare histological subtypes such as neuroendocrine prostate cancer to further define the value of [^18^F]-AlF-NOTA-neurotensin.

## Conclusion

In treatment-naïve prostate cancer, [^68^Ga]Ga-PSMA-617 maintains excellent patient-level sensitivity and lesion detection, outperforming [^18^F]-AlF-NOTA-neurotensin, and should remain the first-line molecular imaging probe. After ADT, [^68^Ga]Ga-PSMA-617 sensitivity and lesion detection decline, whereas [^18^F]-AlF-NOTA-neurotensin significantly reduces missed diagnoses. Therefore, [^68^Ga]Ga-PSMA-617 is preferred before treatment, and addition of [^18^F]-AlF-NOTA-neurotensin or switch to it is recommended after treatment to maximise tumour detection across different clinical stages.

## Supplementary Material

Supplementary Table 1.docx

## Data Availability

The datasets generated during and/or analyzed during the current study are available from the corresponding author upon reasonable request.
