# Supplementary material for: First-in-human evaluation of [18F]-AlF-NOTA-neurotensin for NTSR1-targeted imaging of prostate cancer: a head-to-head comparison with [68Ga]Ga-PSMA-617
Source: Ann Med. 2026 Jun 17;58(1):2678019. doi: 10.1080/07853890.2026.2678019 (PMC13276814; doi:10.1080/07853890.2026.2678019)
Supplement: Supplementary Table 1.docx [file IANN_A_2678019_SM7917.docx]

Supplementary Table 1. Specific Duration of ADT for Each Patient Prior to PET/CT Scans

| **Patient No.** | **Duration of ADT (Months)** |
| --- | --- |
| 1 | 3 |
| 2 | 6 |
| 3 | 4 |
| 4 | 5 |
| 5 | 4 |
| 6 | 3 |
| 7 | 3 |
| 8 | 6 |
| 9 | 5 |
